# The Nitrate Transporter MtNPF6.8 Is a Master Sensor of Nitrate Signal in the Primary Root Tip of *Medicago truncatula*

**DOI:** 10.3389/fpls.2022.832246

**Published:** 2022-03-18

**Authors:** Lili Zang, Łukasz Paweł Tarkowski, Marie-Christine Morère-Le Paven, Michel Zivy, Thierry Balliau, Thibault Clochard, Muriel Bahut, Sandrine Balzergue, Sandra Pelletier, Claudine Landès, Anis M. Limami, Françoise Montrichard

**Affiliations:** ^1^Institut Agro, INRAE, IRHS, SFR QUASAV, Université d’Angers, Angers, France; ^2^PAPPSO, GQE – Le Moulon, INRA, CNRS, AgroParisTech, Université Paris-Saclay, Gif-sur-Yvette, France; ^3^SFR QUASAV, Université d’Angers, Angers, France

**Keywords:** nitrate signaling, nitrate sensor, transcriptomics, proteomics, primary root tip, *Medicago truncatula*, gene ontology, class III peroxidase

## Abstract

Nitrate is not only an essential nutrient for plants, but also a signal involved in plant development. We have previously shown in the model legume *Medicago truncatula*, that the nitrate signal, which restricts primary root growth, is mediated by MtNPF6.8, a nitrate transporter. Nitrate signal also induces changes in reactive oxygen species accumulation in the root tip due to changes in cell wall peroxidase (PODs) activity. Thus, it was interesting to determine the importance of the role of MtNPF6.8 in the regulation of the root growth by nitrate and identify the POD isoforms responsible for the changes in POD activity. For this purpose, we compared in *M. truncatula* a *npf6.8* mutant and nitrate insensitive line deficient in MtNPF6.8 and the corresponding wild and sensitive genotype for their transcriptomic and proteomic responses to nitrate. Interestingly, only 13 transcripts and no protein were differently accumulated in the primary root tip of the *npf6.8-3* mutant line in response to nitrate. The sensitivity of the primary root tip to nitrate appeared therefore to be strongly linked to the integrity of MtNPF6.8 which acts as a master mediator of the nitrate signal involved in the control of the root system architecture. In parallel, 7,259 and 493 genes responded, respectively, at the level of transcripts or proteins in the wild type, 196 genes being identified by both their transcript and protein. By focusing on these 196 genes, a concordance of expression was observed for most of them with 143 genes being up-regulated and 51 being down-regulated at the two gene expression levels. Their ontology analysis uncovered a high enrichment in POD genes, allowing the identification of POD candidates involved in the changes in POD activity previously observed in response to nitrate.

## Introduction

Plants have the ability to respond to changing environmental conditions through phenotypic plasticity. Notably, the root system is able to sense nutrient availability in soil and adapt its development accordingly (Motte et al., 2019). Nitrogen, as a key element required for the synthesis of proteins and nucleic acids, is one of the major nutrients for plant growth and development. It is preferentially assimilated in the form of nitrate (Krapp, 2015), the most abundant nitrogen source in a typical aerobic agricultural soil cultivated with annual crops due to rapid nitrification (Crawford and Forde, 2002; Wang et al., 2012). However, soil nitrate concentration varies locally and greatly in cultivated fields between low and high values (Signora et al., 2001). In legumes, which have the capacity to establish symbiosis with Rhizobia to fix atmospheric nitrogen, the presence of nitrate in soils controls not only the root growth as shown in the model *Medicago truncatula* (Yendrek et al., 2010; Morère-Le Paven et al., 2011), but also symbiosis establishment (Streeter and Wong, 1988; Van Noorden et al., 2016). The presence of nitrate has, however, different effects on these processes. Whereas it improves seedling anchorage through lateral root development, it could compromise symbiosis interaction. Thus, for legume crops, it is important to know how nitrate regulate these processes to find a way to control the sensitivity of legumes to nitrate, ensuring better legume establishment and symbiosis interaction despite the unavoidable variability in nitrate concentration in fields.

We have previously shown in the model legume *M. truncatula* that nitrate, which restricts the primary root growth, acts as a signal perceived by MtNPF6.8, a nitrate transporter (Pellizzaro et al., 2014). We have also shown in R108, a wild and nitrate sensitive genotype, that in the presence of nitrate, there was a decrease in the length of the primary root tip, an increase in the length of lateral roots (LR) as well as a decrease in the distance from LR to primary root cap (Zang et al., 2020). These phenotypic effects of nitrate signaling were abolished in *npf6.8* RNAi mutants and unsensitive lines, showing the determinant role of the nitrate transporter MtNPF6.8 in the perception and transduction of the nitrate signal (Pellizzaro et al., 2014; Zang et al., 2020). More recently, we have demonstrated that the nitrate signal is mediated by a change in accumulation of main reactive oxygen species (ROS) such as ^●^OH (hydroxyl radical) and H_2_O_2_ (hydrogen peroxide) that results from a change in peroxidases of class III (PODs) activity (Zang et al., 2020).

Here, to determine the importance of the role of MtNPF6.8 in the regulation of the root growth by nitrate and identify the PODs involved in the transduction of the nitrate signal in *M. truncatula*, we performed a coupled transcriptomic and proteomic analysis in the primary root tip, the sensory organ involved in the control of root system architecture (Baluška et al., 2010), of R108 and *npf6.8-3*.

## Materials and Methods

### Plant Materials and Growth Conditions

*Medicago truncatula* seeds of R108 and *npf6.8-3* (RNAi line in R108 background) knocked down in the expression of the nitrate transporter MtNPF6.8 were used in this study. After seed germination, seedlings were grown either on N-free MS solution or MS solution supplied with 5 mM nitrate as described by Pellizzaro et al. (2014). Primary root tip and root mature zone were collected from 10 days-old seedlings. Three independent biological repeats were performed.

### Extraction of RNAs and Proteins

Total RNAs and proteins were extracted from primary root tips of R108 and *npf6.8-3* using an improved protocol based on Nucleospin^®^ RNA Plus kit (Macherey-Nagel, Düren, Germany) and Nucleospin^®^ RNA/Protein kit (Macherey-Nagel, Düren, Germany). Frozen root tips were powdered in liquid nitrogen and suspended in lysis buffer containing β-mercaptoethanol. The homogenate was filtered, using a violet-ring Nucleospin^®^ column provided by the RNA Plus kit and centrifuged (11,000 *g*, 1 min). Then, the supernatant was shared in two equal parts and used for RNA or protein isolation. For some experiments, RNAs were also extracted from the primary root mature zone.

RNA quality was checked using an Agilent 2100 Bioanalyzer (Agilent Technologies, Santa Clara, United States). Proteins were treated according to the procedure previously described (Blein-Nicolas et al., 2015). Protein concentration was estimated using Pierce 660 nm Protein Assay (ThermoFisher) before proteomic analysis.

### Microarray Hybridization, Data Extraction and Normalization

**FIGURE 1:**
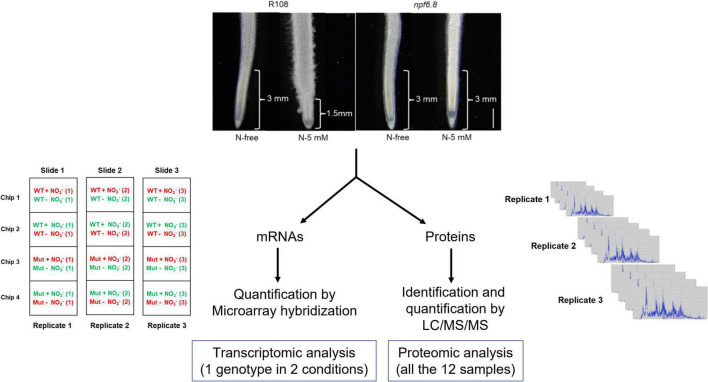
Experimental design for transcriptome and proteome analyses in primary root tip of *M. truncatula*. The upper part of the figure shows representative photographs of root material used for root tip isolation. The approximate length of root tip collected from R108 and *npf6.8-3* seedlings grown in MS without nitrate (N-free) or with 5 mM nitrate (N-5 mM) for 10 days is indicated. Scale bar = 1 mm. For omic studies, three biological replicates were realized, using RNAs and proteins extracted from the same samples. Transcriptomic analysis was performed by co-hybridization on microarrays of cRNAs of R108 (N-free) and R108 (5-mM) or *npf6.8-3* (N-free) and *npf6.8-3* (5-mM), after labeling with cyanine-3 (green fluorescence) or cyanine 5 (red fluorescence). Proteomic analysis was performed in shotgun.

mRNAs were amplified, labeled and hybridized according to the protocol of Celton et al. (2014) with minor modifications. In brief, complementary RNAs (cRNAs) were produced and labelled with Cyanine 3 or Cyanine 5 fluorochromes using the Low Input Quick Amp Labeling Kit (Agilent) from 175 ng of total RNA and purified with RNeasy Mini Kit (Agilent). Labeled samples were mixed as 20 pmol for each dye as shown in Figure 1: R108 (N-free) with R108 (N-5 mM), *npf6.8* (N-free) with *npf6.8* (N-5 mM). Then, they were hybridized for 17 h at 60°C to an Agilent Microarray slide containing 147,454 *M. truncatula* oligomer probes, using the Gene Expression Hybridization Kit (Agilent). The probes were defined from *M. truncatula* genome sequence database Mt5.0 (Pecrix et al., 2018). Afterward, the hybridization and washing were performed according to Agilent Microarray Hybridization Chamber User Guide instructions (©Agilent Technologies, Inc.). A 3 μm resolution scanning on InnoScan 710 scanner (InnopsysR, FRANCE) was performed. The raw data were extracted from the scanned images using MapixR software (InnopsysR, FRANCE) and normalized with the LOESS method. To linearize the gene expression intensity data, they are presented in log2 and the log2 of the ratio is used to compare intensities. Gene expression datasets and microarray design are available *via* the Gene Expression Omnibus (GEO) with identifier GSE197021.

### Real-Time Quantitative PCR

RT-qPCR was performed to validate the transcriptome data. We used for RT-qPCR experiments the same RNA samples as those used for the microarray hybridization. After elimination of genomic DNA using DNase I (Thermo scientific) cDNAs were synthesized from 1.0 μg of RNA using a “iScript Reverse Transcription Supermix” kit (Bio-Rad^®^) according to the manufacturer’s protocol. RT-qPCR was carried out with the Real-Time detection system (Bio-Rad Laboratories, Hercules, CA, United States) using primers designed with Primer Quest Tool^1^ or available in the community of researchers working on *M. truncatula* seedlings. The primers are listed in Supplementary Table 1. The following cycling conditions were applied: initial denaturation at 95°C for 30 s followed by 39 cycles of 95°C denaturation for 10 s, 60°C annealing, 72°C elongation and extension for 20 s. Each reaction mix contained 2 μL previously diluted cDNA (1:2), 5 μL SYBR Green supermix and 100 pmol each primer, for a final volume of 10 μL. The expression of all the genes was determined for each sample and normalized with the expression of two constitutively expressed reference genes: *MtRPB1*, *MtMsc27* (Bouton et al., 2005; Alkhalfioui et al., 2008).

### Protein Digestion, LC-MS/MS Analysis and Protein Identification

Protein digestion and LC-MS/MS analysis were realized according to Blein-Nicolas et al. (2015) with some modifications. Protein identification and filtering were performed by querying MS/MS data against *M. truncatul*a genome Mt5.0 database (Pecrix et al., 2018) together with a custom contaminant database (trypsin, keratins), using X!Tandem Alanine (2017.2.1.4; Craig and Beavis, 2004) and X!Tandem Pipeline 3.4.3 (Langella et al., 2017). Peptides were quantified based on extracted ion chromatogram (XIC) using Masschroq software (v2.2.14^2^; Valot et al., 2011). Peptides shared by two or more proteins were removed and the minimal number of peptides to identify a protein was set to 2. The false discovery rates (FDRs) for proteins and peptides were 0.2 and 0.14%, respectively. Relative protein abundance was calculated and defined as the sum of peptide intensities (abundance) considering only reproducible peptides, specific peptides, and correlated peptides that belong to the same protein. The protein abundance data are presented in log10 and the log10 of the ratio is used to compare intensities. Data are available *via* ProteomeXchange with identifier PXD030547.

### Statistical Analysis

All statistical analyses for transcriptomics and proteomics were performed using the lmFit function and the Bayes moderated *t*-test with the LIMMA package in R software (Smyth, 2004; Ritchie et al., 2015). Transcripts/proteins were considered differentially accumulated if the adjusted *p-value* is under 0.05 based on the false discovery rate procedure of Benjamini and Hochberg (1995).

## Results

### Transcriptomic and Proteomic Analysis Design and Accuracy

For the omic study, three biological replicates were performed and RNAs and proteins were extracted from the same tip samples collected after 10 days of seedling grown without or with 5 mM nitrate (Figure 1). Transcript accumulation was analyzed by microarray hybridization, using a microarray recently designed in our lab that displays probes for 43,825 (upon 44,623) genes coding for proteins known in *M. truncatula* Jemalong, according to the last annotated genome version of this species, Mt5.0 (Pecrix et al., 2018)^3^. Protein identification and accumulation were determined by LC/MS/MS. A total of 3,104 individual proteins were detected in the primary root tips.

**FIGURE 2:**
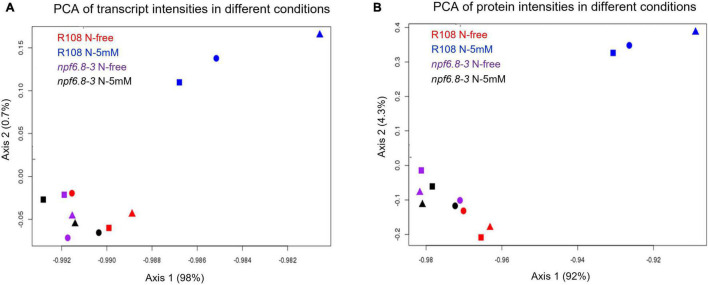
Principal component analysis of transcriptomic and proteomic data. Values of transcripts **(A)** or proteins **(B)** from each biological replicate under the four different conditions are projected onto the first two principal components (Axis 1 and Axis 2). Square, round and triangle correspond to the three independent replicates.

To assess the experimental variability of the data obtained for the two genotypes, in the two nitrate conditions and the three biological replicates, sets of data were compared two by two by plotting the log2 of transcript levels (Supplementary Figures 1A–D) or log10 of protein levels (Supplementary Figures 1E–H) through linear regression analysis. In all cases, the value of the R coefficient is between 0.88 and 1 showing a reliable variability among the biological replicates for both transcriptomics and proteomics. Principal component analysis (PCA) was performed to further assess consistency among the biological replicates (Figure 2). This figure confirms that there is no technical bias, especially replicate bias, in this experiment. For both transcriptomic and proteomic experiment, the main component split samples into two blocks, separating WT-N-5 mM from the others. This means that N-5 mM has a strong effect on the WT but not on the mutant.

**FIGURE 3:**
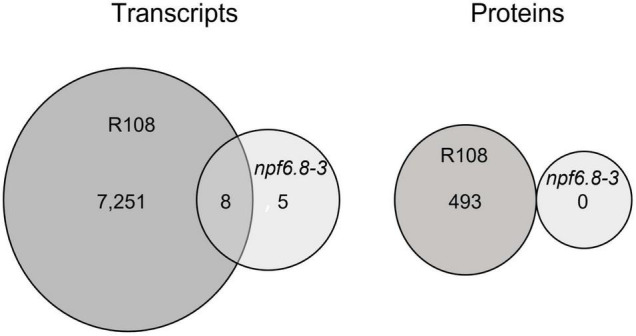
Transcriptomic and proteomic response to nitrate of the primary root tip of R108 and *npf6.8-3*. Venn diagrams show the sets of transcripts and proteins that are differentially accumulated in R108 or *npf6.8-3* in response to 5 mM nitrate and their overlapping. Transcripts and proteins were considered as differentially accumulated if the adjusted *p-value* is under 0.05 based on the false discovery rate procedure of Benjamini and Hochberg (1995).

For an integrative analysis of omic results, transcriptomic and proteomic data were aligned in a unique table (Supplementary Table 2). Levels of transcript and protein abundance are indicated in log2 and log10, respectively. Changes in transcript and protein accumulation induced by 5 mM nitrate were then determined by log ratio on the basis of cutoff values with significantly different expression at *P*-value < 0.05 and false discovery rate (FDR) with the Benjamini-Hochberg correction (BH value < 0.05). In the wild (nitrate-sensitive) genotype, 7,259 genes responded to nitrate at the level of transcripts (16% of the genes on the microarray) and 493 genes responded at the level of proteins (16% of the 3,104 proteins identified in the primary root tip) (Figure 3). In contrast, in the mutant, almost no change in gene expression occurred with only 13 transcripts and no protein differentially accumulating in response to nitrate (Figure 3).

### MtNPF6.8 Is Responsible for Nitrate Sensing in the Primary Root Tip

PCA analysis shows that, whatever the nitrate conditions, the *npf6.8-3* mutant behaves as the wild type grown without nitrate, with a first axis mostly explaining the separation of the R108 sample with N-5 mM from the samples of the three other conditions for both transcriptome (Figure 2A, 98%) and proteome datasets (Figure 2B, 92%). Consistently, almost no change in gene expression occurred in the mutant in the presence of nitrate with only 13 transcripts and no protein differentially accumulated in response to nitrate (Figure 3 and Supplementary Table 3). These genes code a hypothetical protein (*MtrunA17Chr2g0292821*), a putative triacylglycerol lipase (*MtrunA17Chr7g0228261*, a putative ribonuclease T(2) (*MtrunA17Chr5g0417371*), a putative encoded peptide (*MtrunA17Chr8g0374781*), four putative hemopexin-like domain-containing proteins (*MtrunA17Chr6g0468991*, *MtrunA17Chr6g0469031*, *MtrunA17Chr6g0469061* and *MtrunA17Chr6g0469051*), a putative alcohol dehydrogenase (*MtrunA17Chr3g0125961*) and alcohol dehydrogenase 1 (*MtrunA17Chr3g0125911*), a putative pectinesterase (*MtrunA17Chr8g0354911*), a putative SGNH hydrolase-type esterase domain-containing protein (*MtrunA17Chr7g0242461*) and an endochitinase (*MtrunA17Chr8g0370821*). Thus, an obvious link between the functions of those genes and the nitrate treatment is difficult to make unless in the case of the putative pectinesterase, an enzyme well known to be able to act on cell wall mechanical extensibility. Among these genes, eleven are over-expressed and two are under-expressed. Eight of these genes responded to nitrate in the mutant as in the wild type, including the gene of pectinesterase that is up-regulated (Supplementary Tables 2, 3).

**FIGURE 4:**
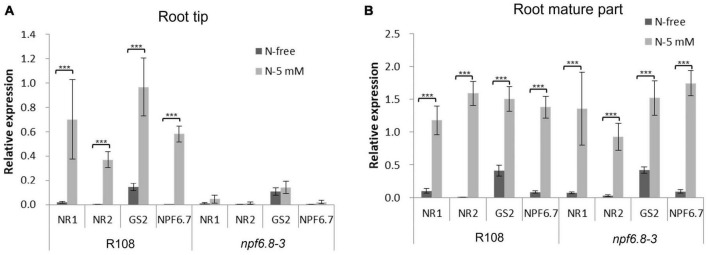
Relative expression of sentinel nitrate-responsive genes in the primary root tip and root mature part of R108 and *npf6.8-3*. RNAs were extracted from R108 or *npf6.8-3* root tip and root mature part of 10 days old seedlings grown in MS without nitrate (N-free) or with 5 mM nitrate (N-5 mM). RT-qPCR experiments were performed with root tip RNAs **(A)** or root mature part RNAs **(B)** using *MtRPB1* and *MtMsc27* as reference genes. *NR1*, nitrate reductase 1; *NR2*, nitrate reductase 2; *GS2*, glutamine synthetase; *NPF6.7*, NRT1/PTR Family 6.7. The statistical test used is an ANOVA (p < 0.001).

We further analyzed by RT-qPCR, a method more sensitive than microarray hybridization, whether nitrate induced an expression of nitrate sentinel genes well known in *M. truncatula* such as nitrate reductases (*NR1* and *NR2*), a glutamine synthetase (*GS2*) and the nitrate transporter 1/peptide transporter family 6.7 (*NPF6.7*), an induction that would be not visible using microarrays. RT-qPCR experiments were performed using the same RNAs as those used for microarrays hybridization (Figure 4A). We also performed the experiment using RNAs extracted from the mature part of the primary root of the mutant. The results were compared with those obtained in the wild type (Figure 4A).

Interestingly, the four sentinel genes were found not to respond in the primary root tip of mutant plants to nitrate but they were highly induced in wild type roots. In the primary root mature part, these genes were overexpressed in the presence of nitrate in the mutant as in the wild type (Figure 4B). Because in the mutant, nitrate has no effect on primary or lateral root growth (Zang et al., 2020), we can conclude that MtNPF6.8 present in the primary root tip confers the nitrate sensitivity of root architecture.

### Changes in Gene Expression Induced by Nitrate in the Wild Type

**FIGURE 5:**
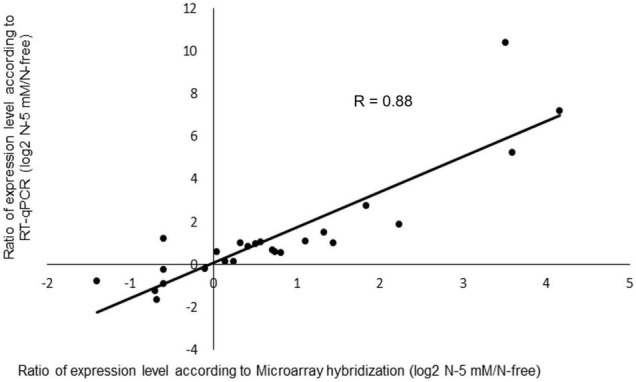
Comparison of differential gene expression in R108 analyzed by microarray hybridization or RT-qPCR. For the experiment, a set of 25 genes, 4 nitrate sentinel genes (*NR1*, *NR2*, *GS2*, *NPF6.7*) and 21 randomly selected genes were used (Supplementary Table 1). Log2 ratio of N-5mM/N-free of gene expression determined by RT-qPCR was plotted as a function of the log2 ratio obtained by microarray hybridization.

Before analyzing the nitrate-responsive genes in the wild type, we performed a validation of the transcriptomic data for this genotype as a whole. For this purpose, RT-qPCR experiments were performed using a set of 25 genes mostly selected randomly. The 25 genes comprise four of the nitrate sentinel genes (*NR1*, *NR2*, *GS2*, *NPF6.7*) mentioned above and 21 other genes having diverse functions (Supplementary Table 1). The log2 ratio of N-5 mM/N-free of gene expression determined by RT-qPCR was further plotted as a function of the log2 ratio obtained by microarray hybridization (Figure 5). The good correlation between these two ratios, with a rather high *R* value (0.88) highlights the general accuracy of our transcriptomic data.

**FIGURE 6:**
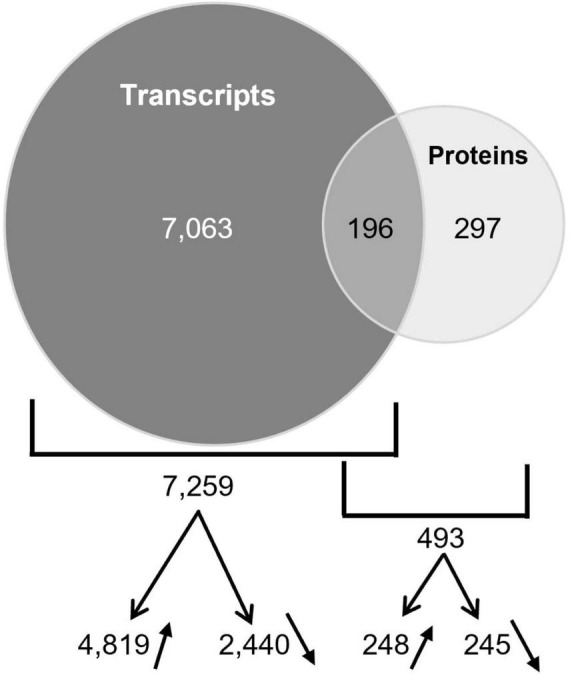
Overlapping of transcripts and proteins differentially accumulated in R108. Venn diagram shows the genes that have a differential expression at the level of transcript only, protein only or both levels. The numbers of transcripts or proteins over- or under-accumulated are also indicated with arrows.

Among the genes responding to nitrate in the wild type, 7,063 genes responded at the level of transcripts only, 297 at the level of proteins only and 196 at both levels (Figure 6). At the level of transcripts, 4,819 and 2,440 are over or under accumulated, respectively. At the level of proteins, 248 and 245 are over or under accumulated, respectively. Altogether these data give a large information about the genes involved in the nitrate response in the primary root tip.

#### Concordance of Expression of the Genes Responding to Nitrate at the Levels of Transcripts and Proteins

The comparative studies at the levels of transcripts and proteins allowed us to identify the genes which expression changed both at transcript and protein level, in response to nitrate. Those genes are likely involved in that response because cognate proteins but not transcripts carry gene functions. Therefore, we decided to focus on the 196 gene set to find functions possibly involved in nitrate response. Interestingly, a concordance of expression was observed for 194 of them, 143 genes being up-regulated and 51 being down-regulated at both levels (Supplementary Table 2). Thus, it was a good opportunity for us to identify genes in this category. The two remaining genes are a putative galacturan 1.4-alpha-galacturonidase and a peroxidase.

**FIGURE 7:**
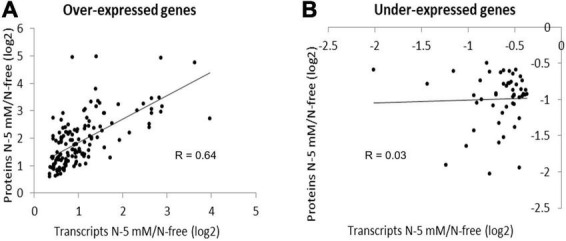
Comparison of transcript and protein level fold-changes in response to nitrate for the 196 differentially expressed genes identified in R108 by their two products (transcript and protein). Log2 ratio of transcript level fold-changes were plotted as a function of the log2 ratio protein level fold-changes for the 143 over-expressed genes **(A)** and the 51 under-expressed genes **(B)**.

To further examine the degree of concordance, log ratios of changes in expression at the transcript level and protein level were compared for each gene (Figure 7). For the transcripts and cognate proteins that are over-accumulated, the factor of correlation is quite high (*R* = 0.64) indicating a strong contribution of the regulation of these genes at the transcriptional level. In contrast, for the transcripts and cognate proteins that are under-accumulated, it is nearly equal to zero (*R* = 0.03). This means that the time-course of the decrease differs between mRNA and protein in response to nitrate. This indicates that additional processes of gene expression regulation intervene in the case of these down regulated genes. Nonetheless, for these 194 genes, a change in transcript accumulation results in a change in protein accumulation. These changes may have a repercussion on cell functions because proteins carry gene functions.

#### Peroxidase Are Major Actors in the Mediation of the Nitrate Signal

**FIGURE 8:**
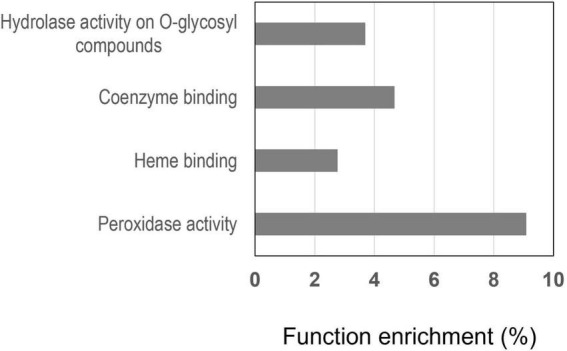
Molecular functions most enriched in the 196 genes in R108 responding to nitrate by both their transcript and protein. The enrichment, expressed in percent, was determined by an agriGO analysis.

To interpret the changes in cell functions induced by nitrate in the primary root tip of the wild type, we performed an agriGO gene ontology (GO) enrichment analysis (Du et al., 2010; Tian et al., 2017) in the 196 common gene set selecting the *M. truncatula* Mt4.0 annotated genome available in agriGO (Lagunas et al., 2019). In the “Molecular function” overview (Supplementary Figure 2), the functions most enriched are: (1) “catalytic activity,” (2) “antioxidant activity” and “oxidoreductase activity” converging on “peroxidase activity,” (3) “tetrapyrrole binding” and “iron binding” converging on “heme binding” (4) “hydrolase activity hydrolyzing *O*-glycosyl compounds,” (5) “coenzyme binding.” The enrichment of the functions “peroxidase activity,” “heme binding,” “hydrolase activity” and “coenzyme binding” is shown in Figure 8. The function “peroxidase activity” with 10 proteins (Table 1) is the first most enriched function (9%) and “heme binding” with 13 proteins is the second most enriched function (3.5%). It is noted that the functions “peroxidase activity” and “heme binding” are overlapping, having in common 9 PODs. This is not surprising because PODs are heme containing enzymes. Eight of these PODs are of class III (Table 1) according to the Peroxibase^4^ or sequence similarity. PODs of class III are involved in ROS metabolism that controls cell elongation or arrest of cell elongation, depending on the zones of the root where they accumulate. This highlights the importance of the role of class III PODs in the response of the root tip to nitrate. These PODs are good candidates to be involved in the changes of peroxidase activity we previously demonstrated (Zang et al., 2020).

**TABLE 1:** Heatmap of nitrate response of peroxidase genes identified in the 196 genes responding to nitrate by both their transcript and protein in R108.

Gene name	Peroxibase	Gene ID (Mt4.0)	Gene ID (Mt5.0)	Transcripts	Proteins
Putative peroxidase	MtPrx33	Medtr7g072480| Medtr7g072490	MtrunA17Chr7g0244451	0.77	0.75
Putative peroxidase	MtPrx46	Medtr5g021060	MtrunA17Chr5g0404721	0.72	0.67
Putative peroxidase	MtPrx48	Medtr6g043240	MtrunA17Chr6g0467121	0.66	0.71
Putative peroxidase	New Prx	Medtr1g054205	MtrunA17Chr1g0175561	0.62	0.56
Catalase-1/2	MtKat01	Medtr3g115370	MtrunA17Chr3g0143911	0.61	0.36
Putative phospholipid-hydroperoxide glutathione peroxidase	MtGPx02	Medtr8g105630	MtrunA17Chr8g0391921	0.52	0.38
Putative peroxidase	MtPrx12	Medtr2g029815	MtrunA17Chr2g0292211	0.48	0.19
Putative peroxidase	MtPrx34	Medtr3g072190	MtrunA17Chr3g0114451	0.43	0.82
Putative peroxidase	MtPrx14	Medtr4g127670	MtrunA17Chr4g0072091	−0.49	0.18
Putative peroxidase	MtPrx60	Medtr2g099175	MtrunA17Chr2g0329641	−0.63	−0.36

*Gene names, gene IDs (Mt4.0 and Mt5.0) and changes in expression (N-5 mM/N-free) in log2 ratio for transcripts and log10 ratio for proteins are indicated (red for up-regulation and green for down-regulation). Data were extracted from the Supplementary Table 2.*

The enrichment in hydrolases active on *O*-glycosyl bonds (10 proteins) is not surprising (Figure 8) since most enzymes in this category are involved in the metabolism of cell wall polysaccharides, polymers that play a role in the process of cell elongation or restriction of elongation.

## Discussion

In this study we performed an integrated omic study in the tip of the primary root of *M. truncatula* to determine the importance of the role of MtNPF6.8 in the nitrate signaling pathway and identify the PODs involved in the transduction of the nitrate signal.

### MtNPF6.8 Is a Master Sensor of Nitrate at the Primary Root Tip Level

Although nitrate signaling has been investigated for many years, in different organs and at different omic levels, including transcriptomics, proteomics and metabolomics (Wang et al., 2000, 2001, 2003; Krouk et al., 2010; Ruffel et al., 2011; Cabeza et al., 2014; Menz et al., 2016; Vicente et al., 2016), few studies have focused on the primary root tip. However, there is evidence supporting a sensory role for the primary root tip for exogenous nutrient concentrations and other environmental cues (Baluška et al., 2010; Sahu et al., 2020). For instance, the response to phosphorus supply requires a physical contact between the root tip and the low-Pi in the medium. Another interesting case is exogenous *L*-glutamate (Glu) that slows down primary root growth and stimulates root branching in *A. thaliana*. It was found that *L*-Glu is sensed at the primary root tip (Walch-Liu et al., 2006). Furthermore, the existence of a glutamate signaling pathway starting at the root tip is supported by the discovery in plants of a family of GLR receptors (glutamate-like receptors, homologs of ionotropic glutamate receptors found in vertebrates) and the finding that the MEKK1 (mitogen-activated protein kinase kinase kinase 1) gene is a positive regulator of glutamate sensitivity at the root tip (Forde et al., 2013).

Although nitrate was shown to act as a signal to modulate root system architecture, an action of nitrate as a signaling molecule specifically at the root tip level is awaiting more evidence to be strengthened. In *A. thaliana* such an effect of nitrate was shown only in conjunction with glutamate. Nitrate was able to stimulate primary root growth by antagonizing the inhibitory effect of glutamate (Walch-Liu and Forde, 2008). The response to nitrate was not mimicked by ammonium (NH_4_^+^) as an alternative N source and depended on a direct contact between the primary root tip and nitrate. Furthermore, the mutant defective in the nitrate transporter AtNPF6.3 (*chl1-5*) was insensitive to nitrate antagonizing effect on glutamate signaling (Walch-Liu and Forde, 2008). Thus, to our knowledge, the present study is the first one to bring together phenotypic, transcriptomic and proteomic data strongly in favor of the perception of nitrate as a signaling molecule at the primary root tip level with an emphasis on the crucial role of MtNPF6.8 in the perception of this signal. The nearly total absence of response of the mutant to nitrate at the level of transcripts and proteins support this assertion and shows that the integrity of MtNPF6.8 is required for the sensitivity of the primary root tip to nitrate. Thus, we propose that MtNPF6.8 functions as a master nitrate sensor in the root tip, governing the expression of most nitrate inducible genes in this zone. Also, on the basis of phenotypic data, it appears that nitrate sensing by MtNPF6.8 at the level of the root tip is necessary for the response of whole root system architecture to nitrate.

Induction of the sentinel genes by nitrate in the mature zone of the root in the mutant supports the idea that a MtNPF6.8-independent perception system of nitrate signal might exist in *M. truncatula* root. This system might be involved in other processes such as response of the root to nitrate-rich patches or nodulation. However, since the phenotypic response to nitrate was abolished in the mature zone (phenotype of LR) in the absence of a functional MtNPF6.8 (Zang et al., 2020) we propose that, at least under homogenous nitrate supply, the control of the whole root system architecture by nitrate might be governed at the root tip level by MtNPF6.8. This suggestion is consistent with the supposed role of the root tip in exploring the soil for nutrients (Baluška et al., 2010). Regarding the very few genes, thirteen, induced by nitrate in the root tip of the mutant, they might be controlled by an alternative system. Nevertheless, in the absence of further evidence, we can’t rule out that they are due to a contamination of the tip fraction by the mature zone fraction.

Expression of *MtNPF6.8* is rather low in the tip and is much lower than in the mature part of the root as we have previously shown by RT-qPCR (Pellizzaro et al., 2014). Moreover, it appears here not to vary significantly in the tip in response to nitrate. The cognate protein was not detected in the proteome, indicating that the transporter is a low abundant protein in the primary root tip. However, consistently with its signaling role in the root tip, the low abundance of MtNPF6.8 protein does not affect its action as it probably intervenes as the first player in the signaling cascade with partners responsible of the amplification of the signal. The low abundance of MtNPF6.8 is probably also a consequence of the fact that only a tiny part of the root tip, namely the transition zone, functions as a sensory center able to translate environmental information into developmental response (Baluška et al., 2010).

### Identification of Peroxidase Candidates That Mediate the Nitrate Signal

**FIGURE 9:**
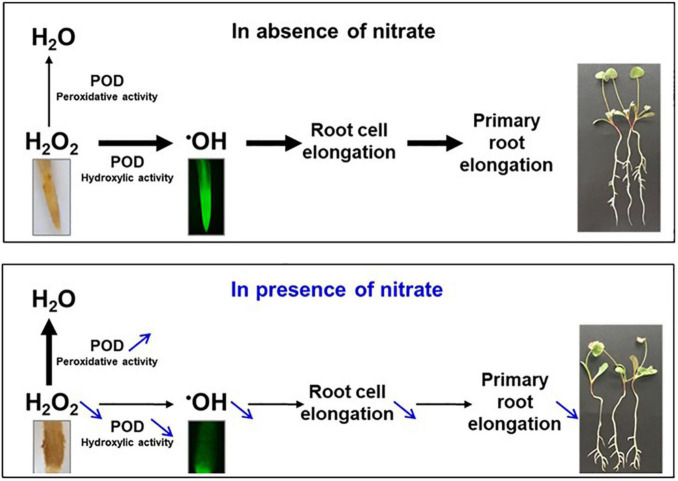
ROS and POD govern primary root growth in *M. truncalula*. In absence of nitrate **(upper panel)**, H_2_O_2_ accumulated in the primary root tip is for a large part converted by POD hydroxylic activity in ^●^OH, a ROS that favors root cell elongation, and root elongation, through cell wall polymer breaking. H_2_O_2_ is also for a smaller part eliminated by POD peroxidative activity, resulting in H_2_O. In the presence of nitrate **(lower panel)**, both the H_2_O_2_ abundance and the POD hydroxylic activity decrease while POD peroxidative activity increases. As a result, ^●^OH is less produced, restricting cell and root elongation. H_2_O_2_ and ^●^OH were detected in the root tip with diaminobenzidine (yellow color) and dihydrorhodamine (green fluorescence), respectively. 10-day old seedlings grown in the absence or the presence of 5 mM nitrate are presented at the right of the panels. Adapted from the graphical abstract of Zang et al. (2020).

In *M. truncatula*, we have shown that the restriction of the primary root growth induced by nitrate was due to a restriction of cell elongation (Pellizzaro et al., 2014). We have further demonstrated that the nitrate signal is sensed by the nitrate transporter MtNPF6.8 (Pellizzaro et al., 2014) and transduced by changes in ROS accumulation, in particular for H_2_O_2_. This is not surprising because ROS are well known to govern root growth and their levels are regulated in case of stress, as recently reviewed in Considine and Foyer (2021). In *M. truncatula*, changes in H_2_O_2_ accumulation is due to changes in POD activity in the root tip (Zang et al., 2020). PODs present in cell wall and apoplast of root cells are versatile enzymes (Passardi et al., 2004, 2005) that were shown to govern root growth (Liszkay et al., 2003; Dunand et al., 2007; Tsukagoshi et al., 2010). Indeed, in root elongation zone, H_2_O_2_ is converted by POD hydroxylic activity in ^●^OH, a ROS that breaks cell wall polymers and allows cell elongation. In contrast, in the root differentiation zone, H_2_O_2_ accumulates and restricts cell elongation by cell wall polymer cross linking (Eljebbawi et al., 2021). In *M. truncatula*, we recently demonstrated that nitrate induced a decrease in H_2_O_2_ abundance, a decrease in POD hydroxylic activity and an increase in POD peroxidative activity in the primary root tip (Figure 9). We have further shown that an addition in the growth medium of H_2_O_2_ counteracts the effect of nitrate while the addition of KI, an H_2_O_2_ scavenger, mimics a nitrate effect (Zang et al., 2020). Similar results were also obtained in *Arabidopsis thaliana* by Chu et al. (2021). All these results indicate that H_2_O_2_ and PODs play a crucial role in the root growth response to nitrate. We observed here among the genes responding to nitrate at both levels of transcript and protein an enrichment of class III POD genes that confirms the involvement of these PODs in the response to nitrate as we have previously demonstrated (Zang et al., 2020). The importance of PODs in the response to nitrate is also well visible in the whole omic results through the high percentage of enrichment calculated for class III POD genes (about 50%, Supplementary Table 4) with 65 genes being up or down regulated in presence of nitrate over 119 genes found in the last version of the genome (Mt5.0) of the species (PeroxiBase, personal data). Trevisan et al. (2015) working on maize root apex came to the same conclusion that PODs play a determinant role in the root sensitivity to nitrate. These results are also consistent with a role of PODs in root nutrient responses. Thereby, POD genes expressed in the primary root tip were shown to be involved in the root response to other nutrient signals such as phosphate signal. A combined transcriptomic and proteomic analysis performed on phosphate-starved root tips of *A. thaliana* revealed that more than 30 different PODs were affected by Pi starvation at the transcript or protein level (Hoehenwarter et al., 2016). In this species, POD peroxidative activity response to Pi starvation was proposed to generate crosslinks in the cell wall polymers, eventually resulting in cell wall stiffening and root growth arrest (Balzergue et al., 2017). PODs were shown to cover important roles also in the potassium starvation response. Research on *A. thaliana* roots showed that the POD gene *AtRCI3* (Rare Cold response-Induced) is induced by potassium deficiency and its expression was found to be required for the subsequent expression of the high-affinity potassium transporter *AtHAK5* (Kim et al., 2010).

In *M. truncatula*, as already mentioned above, the restriction of the primary root growth was shown to be accompanied by an increase in POD peroxidative activity and a decrease in POD hydroxylic activity in the primary root tip (Zang et al., 2020), both changes explaining the decrease in ^●^OH production, an effect that is probably responsible of the restriction of cell elongation. Thus, among the POD genes that respond to nitrate identified here, those responsible of these changes may be present (Table 1). Because the expression of POD genes common to the differential transcriptomics and proteomics is rather concordant, a way to regulate POD activity could be directly achieved by a regulation of POD abundance. Thus, we hypothesize that MtPrx60, the only POD that is under-accumulated in the presence of nitrate, is a good candidate to have an hydroxylic activity that decreases in the presence of nitrate whereas PODs that are over-accumulated (MtPrx12, 14, 33, 34, 46, 48, and a new Prx) are good candidates to have a peroxidative activity that increases in the presence of nitrate. We are currently investigating in these directions.

In *A. thaliana*, POD genes involved in root elongation were shown to be under negative control of UPBEAT1 transcription factor (Tsukagoshi et al., 2010). A putative ortholog to UPBEAT1 exists in *M. truncatula* (MtrunA17Chr1g0200631) that may have the same function. Its gene was under-expressed in the primary root tip in the presence of nitrate (Supplementary Table 2). Thus, the up-regulation observed for some POD genes in *M. truncatula* could be due to a down-regulation of this gene.

On top of changes in protein abundance, changes in protein activity through protein modification, for example by protein kinases, cannot nevertheless be ruled out. This is supported by the high enrichment of this function in the R108 transcriptome in response to nitrate (Supplementary Table 2) even if this function is not enriched in the proteome. The absence of detection of protein kinases in the proteome is likely due to the fact that protein kinases are low abundant proteins. Whether protein kinases control POD activity or/and other proteins intervening in the nitrate signaling pathway remains to be determined.

## Conclusion and Perspectives

The most important result of this study is the discovery that MtNPF6.8 plays a master role in the nitrate signal sensing in the primary root tip of *M. truncatula* that determines the whole root architecture response to nitrate. This finding has potential breeding applications for legume crops. Whether a manipulation of nitrate sensitivity could improve legume seedling homogeneity and ability to capture soil nitrate remained to be determined. Solving this question is crucially important because legume seedlings optimized for nitrate signaling and utilization would benefit to both environment and yields.

## Data Availability Statement

The data presented in the study are deposited in Gene Expression Omnibus (accession number GSE197021) and ProteomeXchange (accession number PXD030547).

## Author Contributions

LZ: biological material production, RNA and protein extraction, RT-qPCR, transcriptome and proteome analysis and interpretation, and writing of the manuscript. ŁT: transcriptome and proteome analysis and interpretation and writing of the manuscript. FM, M-CM-LP, and AL: conception and design of the work and acquisition, analysis, and interpretation of data for the work, and writing of the manuscript. TC: biological material production, RNA and protein extraction, RT-qPCR. MZ and TB: proteomic expertise, proteomic data production, and statistical analysis. MB, SB, SP, and CL: conception of the novel *M. truncatula* microarray, based on Mt05, transcriptomic expertise, supervision of transcriptomic data production, and statistical analysis. All authors contributed to the article and approved the submitted version.

## Conflict of Interest

The authors declare that the research was conducted in the absence of any commercial or financial relationships that could be construed as a potential conflict of interest.

## Publisher’s Note

All claims expressed in this article are solely those of the authors and do not necessarily represent those of their affiliated organizations, or those of the publisher, the editors and the reviewers. Any product that may be evaluated in this article, or claim that may be made by its manufacturer, is not guaranteed or endorsed by the publisher.

## Footnotes

1
https://eu.idtdna.com/Primerquest/Home/Index

2
pappso.inra.fr/bioinfo/masschroq/

3
https://medicago.toulouse.inra.fr/MtrunA17r5.0-ANR/

4
https://peroxibase.toulouse.inra.fr/
