# Supplementary material for: The Nitrate Transporter MtNPF6.8 Is a Master Sensor of Nitrate Signal in the Primary Root Tip of Medicago truncatula
Source: Front Plant Sci. 2022 Mar 18;13:832246. doi: 10.3389/fpls.2022.832246 (PMC8971838; doi:10.3389/fpls.2022.832246)
Supplement: Supplementary file 1 [file Data_Sheet_1.PDF]

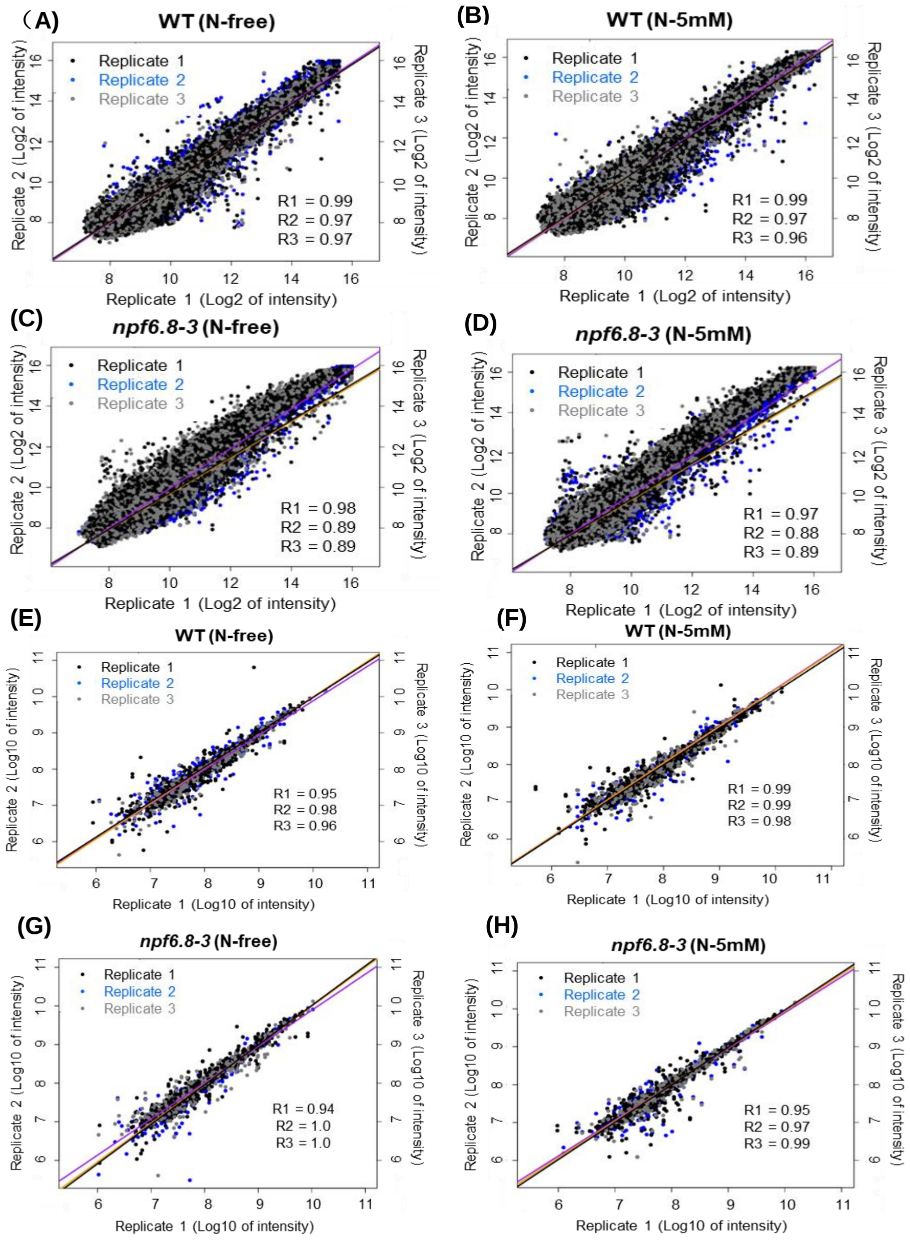

Figure S1. Analysis of data variability in the three biological replicates. (A-D) Gene expression data in transcriptomics; (A) R108 (N-free), (B) R108 (N-5 mM), (C) *npf6.8-3* (N-free) and (D) *npf6.8-3* (N-5 mM). (E-H) Protein accumulation data in proteomics; (E) R108 (N-free), (F) R108 (N-5 mM), (G) *npf6.8-3* (N-free) and (H) *npf6.8-3* (N-5 mM). The levels of gene expression or protein accumulation are provided in a log2 or log10 scales. The experimental data corresponding to genes or proteins detected are spotted in black (replicate 1), blue (replicate 2) or grey (replicate 3). The linear regression lines and the correlation coefficients R are provided for comparison of replicate 1 with replicate 2 (purple), replicate 1 with replicate 3 (yellow) and replicate 2 with replicate 3 (black).

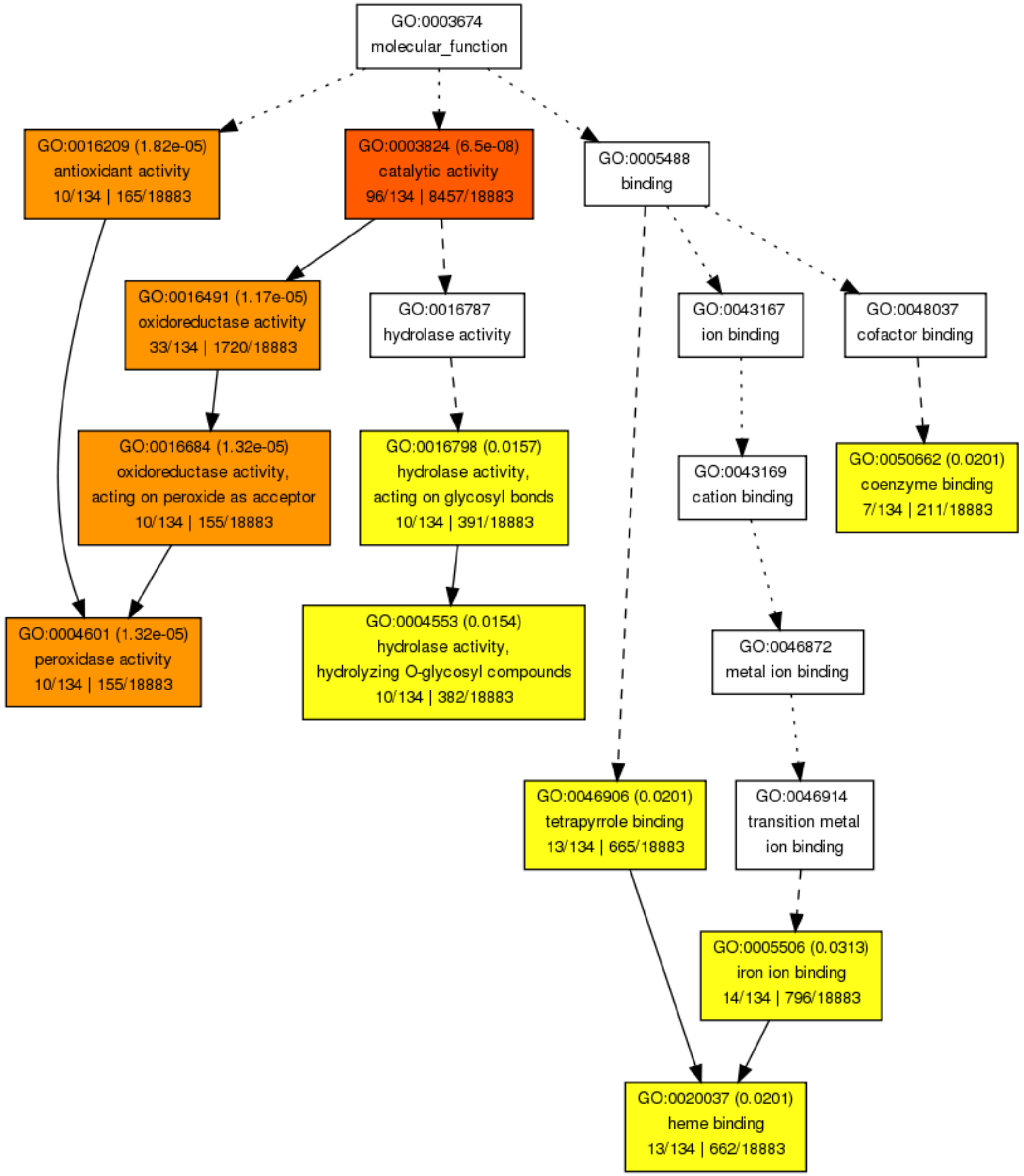

Figure S2. Molecular functions enriched in the 196 genes responding to nitrate by both their transcript and protein as determined by an Agrigo analysis.

Table S1. List of primer sequences used for RT-qPCR experiments.

	Gene ID (Mt5.0)	Gene name	Primers	
1	MtrunA17Chr3g0115151	Nitrate reductase 1 (*NR1*)	Forward	TGGCCCCCATCAAGGAA
			Reverse	CACGCGGGTTTAAAGCTACAC
2	MtrunA17Chr5g0424491	Nitrate reductase 2 (*NR2*)	Forward	TTCCATTGGCTGGTGAGGAT
			Reverse	GGTTGCACAGCAAATTGAATCA
3	MtrunA17Chr2g0287181	Glutamine synthetase (*GS2*)	Forward	GTTATTGGTTATGAGATGAATGCACAT
			Reverse	TGCAACTCTGTCCATACCATATGC
4	MtrunA17Chr5g0399071	Nitrate transporter 1/peptide transporter family (*NPF6.7*)	Forward	GTGACCGGTCCAAATAAACCATGG
			Reverse	ACCCCTATGCATGGAATGTAGAGGC
5	MtrunA17Chr2g0283471	Pyruvate decarboxylase 1	Forward	GCGTTCGCGACGTCTTCT
			Reverse	CTCAGCGATCAGGTGGTCAA
6	MtrunA17Chr5g0399061	Nitrate transporter 1/peptide transporter family (*NPF6.6*)	Forward	GAAAGTGCTGGTGGAATCGT
			Reverse	CCATATAGGGAGCATTCTTAGC
7	MtrunA17Chr2g0322861	Peroxidase (*Prx05*)	Forward	AAAGCTCTAGTGACTCAATTCC
			Reverse	CCTTGTGACCCTGTCTTAACT
8	MtrunA17Chr4g0024121	Peroxidase (*Prx53*)	Forward	CCATTGGTGGGCCTTATT
			Reverse	GGACAGTGAGGTTGTGAAA
9	MtrunA17Chr1g0195151	Putative transcription factor AP2-EREBP family	Forward	TGCCACCTAATAATGTTCAGGA
			Reverse	TTCACAGAAGAAACCGAAGCA
10	MtrunA17Chr8g0385931	Putative beta-galactoside alpha-2.3-sialyltransferase	Forward	CAAGCACTGAGAGATAGCAGCA
			Reverse	TCAAAGAGAAGCAACACAAGGTC
11	MtrunA17Chr3g0090681	Ribulose bisphosphate carboxylase large chain	Forward	CATGGACAACTGTGTGGACCA
			Reverse	TCTTCTCCAGCAACAGGTTCG
12	MtrunA17Chr2g0295421	Putative transcription factor AP2-EREBP family	Forward	TCTTCCAAAGAGCTACCACC
			Reverse	TTCTTACCTTTCTCCACAGTTACC
13	MtrunA17Chr7g0242961	Putative pyruvate decarboxylase	Forward	TGTTGGTGCAACTCTTGGCTAT
			Reverse	CATCACCAATGCAAGCAATCA
14	MtrunA17Chr7g0266211	Putative transcription factor bZIP family	Forward	AGAGCCAGAAAACAGGCCTATAC
			Reverse	CCTCTCGAGTTCAGCCAGTG
15	MtrunA17Chr3g0098811	Putative peptide-methionine (S)-S-oxide reductase	Forward	AGGTCAACAGTTTGCCCAGT
			Reverse	ACGCATCAAGCAGAGTCTCA
16	MtrunA17Chr6g0462031	Putative chlorophyll A-B binding protein	Forward	TGGCATTTGTTGGAATCGTTGT
			Reverse	ATGTTGTTGTGCCATGGATCAG
17	MtrunA17Chr2g0278591	Putative Actin family	Forward	GCTGACCGTATGAGCAAGGA
			Reverse	TGCCAAGATAGACCCACCAA
18	MtrunA17Chr6g0485711	Putative chlorophyll(ide) b reductase	Forward	TCTGACAGCCGTCTATGGTTC
			Reverse	CGGGAGATGCAGTATGGACA
19	MtrunA17Chr4g0027961	Putative pheophorbide a oxygenase	Forward	GTGTCTCAGGGGTTGCTCTT
			Reverse	AAAGTCATCAGGCAACATTGGA
20	MtrunA17Chr3g0117141	Galactinol--sucrose galactosyltransferase	Forward	AACTCTTGTTTTGCCTGATGGT
			Reverse	AAAACACCCGTGTATTTGTTGAGA
21	MtrunA17Chr2g0288531	Putative autophagy protein Atg8 ubiquitin	Forward	CAGTGGAGAGAACACCTTTG
			Reverse	ACGCATTTGCCATGTATTTG
22	MtrunA17Chr3g0090521	Putative chlorophyllase	Forward	CGAACAAGGTGCCGATTT
			Reverse	CAGCAGGGCTGATTTGATAATA
23	MtrunA17Chr5g0446771	Putative Red chlorophyll catabolite reductase	Forward	GATGAAGGAGAGCGGATAGA
			Reverse	GGCACAATGATCCAACCATA
24	MtrunA17Chr2g0334031	Putative transcription factor AP2-EREBP family	Forward	ACGCCCAATGTGCTAATG
			Reverse	ATTGCTCGAATACTCCGAACT
25	MtrunA17Chr8g0364801	Putative transcription factor Hap3/NF-YB family	Forward	GCGGAGTGAAGAAGCCACA
			Reverse	CACGAACAAGACGCTGGAAA

Table S2. Transcriptomic and proteomic data.

The page "Align omic data" shows an alignment in a unique table of all the data of transcriptomic and proteomic obtained with R108 and the mutant *npf6.8-3*. The values are expressed in log2 for transcriptomics and log10 for proteomics. Changes in transcript and protein accumulation induced by 5 mM nitrate were then determined by log ratio (WT/WTN). Transcripts or proteins were considered differentially accumulated if the adjusted *p-value* is under 0.05 based on the false discovery rate procedure of Benjamini and Hochberg (Benjamini and Hochberg, 1995). The page "196 genes" shows only the data corresponding to the 196 genes responding to nitrate by both their transcript and protein in R108.

Table S2 is provided as an excell file

Gene name	Gene ID (Mt5.0)	WT (N-5mM/N-free)	*npf6.8* (N-5mM/N-free)
Endochitinase	MtrunA17Chr8g0370821	3,37	1,35
Putative SGNH hydrolase-type esterase domain-containing protein	MtrunA17Chr7g0242461	1,94	1,26
Putative pectinesterase	MtrunA17Chr8g0354911	1,37	1,13
Alcohol dehydrogenase 1	MtrunA17Chr3g0125911	1,28	1,28
Putative alcohol dehydrogenase	MtrunA17Chr3g0125961	1,19	0,8
Putative Hemopexin-like domain-containing protein	MtrunA17Chr6g0469061	0,65	1,12
Putative ribonuclease T(2)	MtrunA17Chr5g0417371	0	0,79
Putative encoded peptide	MtrunA17Chr8g0374781	0	0,93
Putative Hemopexin-like domain-containing protein	MtrunA17Chr6g0469051	0	0,97
Putative Hemopexin-like domain-containing protein	MtrunA17Chr6g0469031	0	1,38
Putative Hemopexin-like domain-containing protein	MtrunA17Chr6g0468991	0	1,47
Putative triacylglycerol lipase	MtrunA17Chr7g0228261	-1,23	-1,03
hypothetical protein	MtrunA17Chr2g0292821	-2,75	-1,03

Table S3. Genes responding to nitrate in the *npf6.8*-3 at the transcript level. Data were extracted from the supplemental table S2. Gene names, ID (Mt5.0) and changes in expression in log2 ratio (N-5 mM/N-free) are indicated (red for up-regulation and green for down-regulation).

Transcriptomic data		Proteomic data
0	MtPrx01	0,78
0	MtPrx05	0,41
0,72	MtPrx07	NA
-0,39	MtPrx08	NA
3,16	MtPrx09	NA
0	MtPrx10	0,39
0	MtPrx11	0,22
0,48	MtPrx12	0,19
0,44	MtPrx13	NA
-0,49	MtPrx14	0,18
0	MtPrx15	0,49
0	MtPrx16	0,34
1,31	MtPrx17	NA
-0,56	MtPrx18	NA
1,05	MtPrx19	NA
1,75	MtPrx20	NA
0,69	MtPrx21	NA
2,19	MtPrx22	NA
-0,78	MtPrx24	NA
0,78	MtPrx26	NA
2,61	MtPrx27	NA
2,1	MtPrx28	0,00
0	MtPrx29	0,52
0,77	MtPrx33	0,75
0,43	MtPrx34	0,82
2,47	MtPrx35	NA
3,87	MtPrx36	NA
1,39	MtPrx37	NA
0	MtPrx38	0,37
0	MtPrx39	0,21
4,03	MtPrx41	NA
0,47	MtPrx42	NA
2,53	MtPrx43	NA
0	MtPrx44	0,25
0,72	MtPrx46	0,67
0	MtPrx47	-0,09
0,66	MtPrx48	0,71
2,63	MtPrx49	NA
2,54	MtPrx50	NA
1,34	MtPrx52	NA
0	MtPrx53	0,33
-0,92	MtPrx55	NA
0,88	MtPrx56	NA
0,63	MtPrx57	NA
0,8	MtPrx58	NA
-0,63	MtPrx60	-0,36
2,56	MtPrx66	NA
-0,53	MtPrx76	NA
-0,61	MtPrx79	NA
1,02	MtPrx81	NA
0,63	MtPrx84	NA
0,63	MtPrx86	NA
0,81	MtPrx90	NA
0,51	MtPrx94	NA
0,76	MtPrx98	NA
-0,82	MtPrx107 / MtPrx114	NA
-0,74	MtPrx110	NA
4,08	MtPrx111	NA
0,76	MtrunA17Chr3g0106921	NA
0,62	MtrunA17Chr1g0175561	0,56
0,64	MtrunA17Chr1g0181481	NA
1,22	MtrunA17Chr1g0181511	NA
-0,46	MtrunA17Chr2g0316191	NA
0,76	MtPrx(P)06	NA
0,43	MtPrx(P)113	NA

Table S4. Heatmap of nitrate response of class III peroxidases identified in both transcriptomics and proteomics in R108. Data were extracted from the supplemental table S2.
